# Evaluation of the association between asthma and non-neurogenic urinary incontinence in children; a case-control study

**DOI:** 10.1186/s12887-023-03958-7

**Published:** 2023-03-30

**Authors:** Elaheh Ziaei, Fatemeh Dorreh, Parsa Yousefichaijan, Roham Sarmadian, Nooshin Sajjadi, Manijeh Kahbazi

**Affiliations:** 1 Faculty of medicine, Arak University of Medical Sciences, Arak, Iran; 2 Department of Pediatrics, Arak university of medical sciences, Arak, Iran; 3 Infectious disease research center(IDRC), Arak university of medical sciences, Arak, Iran

**Keywords:** Asthma, Urinary incontinence, Nocturnal enuresis, Overactive bladder, Giggle incontinence, pollakiuria

## Abstract

**Background:**

Asthma is the most common chronic disease in children. Asthma can lead to sleep disorders and psychiatric issues, which are often accompanied by urinary incontinence in children. Furthermore, several studies have shown a relationship between allergic diseases and urinary incontinence. This study aims to examine the association between asthma and non-neurogenic urinary incontinence.

**Materials and methods:**

This case-control study included 314 children over three years old referred to Amir Kabir Hospital; 157 with asthma and 157 without asthma. After explaining each urinary disorder in accordace with the International Children’s Continence Society’s definitions, parents and children were asked about their presence. The disorders included monosymptomatic nocturnal enuresis(MNE), nonmonosymptomatic nocturnal enuresis (NMNE), vaginal reflux (VR), pollakiuria, infrequent voiding, giggle incontinence (GI), and overactive bladder (OAB). The analysis was performed using Stata 16.

**Results:**

The average age of the children was 8.19 ± 3.15 years. Patients with asthma (p = 0.0001) and GI (p = 0.027) had a considerably lower average age than patients without these disorders. Asthma and urinary incontinence, including NMNE, Infrequent voiding, and OAB, were significantly correlated (p = 0.017, 0.013, and 0.0001, respectively). Moreover, the association between MNE and asthma was significant in males (p = 0.047).

**Conclusion:**

Due to the relationship between asthma and urinary incontinence, children with asthma must be evaluated for the presence of urinary disorders and, if present, receive the proper treatment in order to improve their quality of life.

## Background

Asthma is the most common chronic respiratory disease in children worldwide, and if it is not properly controlled, it can lead to stunted growth, higher healthcare costs, and even increased mortality [1]. The quantity and quality of a child’s sleep can be negatively impacted by diseases such as asthma, which frequently cause nocturnal symptoms. Asthma is associated with poor sleep quality, even in children with persistent and well-controlled symptoms [2].

Non-neurogenic urinary incontinence (UI) is the second most common condition in childhood, after allergic diseases, and is described as the unintentional loss of urine that cannot be attributed to any other organic cause or medicine [3]. Bladder and bowel dysfunction can often lead to this condition, and vice versa [4].Children acquire consistent bladder control at about 3 years old, first during the daytime and later at night [5; 6]. Wetting is pathologic in children who are old enough to control their bladder. UI in children is associated with emotional discomfort, behavioral issues, and diminished quality of life [7]. Two distinct forms of UI can be identified by the time they manifest: nocturnal enuresis, which occurs at night while sleeping, and daytime UI, though a combination of both types is also possible [8]. There are several urinary disorders associated with UI.

UI can be associated with several comorbidities. Constipation has been shown to have a strong association with UI in children. Studies show that up to 40% of children with UI also have constipation [9]. UI in children can also be linked to respiratory disorders such as sleep apnea. Children with sleep apnea are more likely to experience UI, and treating it can improve bedwetting [10]. In addition to physical comorbidities, UI has been also associated with psychological and psychiatric disorders like attention-deficit/hyperactivity disorder (ADHD), anxiety, and depression [11; 12].

Since asthma may cause sleep-related breathing problems [13], and since UI, particularly nocturnal enuresis, is associated with sleep-related breathing disorders [14], a relationship can be found between UI and asthma. In addition, asthma is a chronic condition in children, and most chronic diseases in children are associated with psychiatric disorders such as ADHD, depression, and anxiety [15]. Because most psychiatricl illnesses are correlated with UI [11; 16], it is conceivable that asthma is also associated with UI. On the other hand, several studies show a connection between allergic diseases and UI [17; 18]. The goal of this study is to evaluate the association between asthma and urinary disorders associated with urinary incontinence so that, if a relationship is found, children with asthma can be screened for these conditions and given treatment.

## Methods

This research was a case-control study with 314 children over three years old, including 157 children with asthma and 157 children without asthma or a history of asthma symptoms who were referred to the pediatric clinic of Amir Kabir Hospital. Parents of children who visited were questioned about their child’s asthma symptoms and history. If a child exhibits asthma symptoms, such as wheezing in the lungs, cough, and shortness of breath, as well as a history of using inhaler sprays, the diagnosis of asthma is made after the confirmation of pediatric asthma and allergy specialist and the child is then included in the case group. The control group participants were randomly selected from children over three years old with no symptoms of asthma or allergies, a personal or family history of asthma or any other respiratory diseases which mimic asthma symptoms, as well as a history of anatomical disorders in Kidneys and urinary tracts, or neurogenic disorders. After receiving the consent of the child’s parents to participate in the study, the types of urinary disorders, symptoms, and features of each disorder were explained. All disorders and their symptoms were thoroughly described to the patients and their parents based on the International Children’s Continence Society’s definitions [19]. Then the parents or child were questioned about the presence of each disorder. The urinary disorders included MNE and NMNE, VR, pollakiuria, infrequent voiding, GI, and OAB.

MNE is a condition in which a child or adult experiences involuntary urination during sleep at least twice a week, without any other urinary symptoms during the day or any other medical conditions [20]. Nonmonosymptomatic nocturnal enuresis (NMNE) consists of nocturnal symptoms along with lower urinary tract symptoms, such as urgency, changes in voiding frequency, and holding maneuvers, with or without daytime incontinence [21]. Nocturnal enuresis with any severity (mild: less than once a day or less than 7 times a week, moderate: more than once a day or 8–20 times a week, severe: more than 20 times a week [22]) was considered positive. Giggle incontinence (GI) is described by total or involuntary bladder emptying while giggling or laughing, with normal bladder function at other times [19]. vaginal reflux (VR) or intravaginal urination is one of the causes of UI in prepubescent girls, which is accompanied by post-void dribbling, vulvovaginitis, vaginal discharge and foul smell around the vagina [23]. Pollakiuria is defined by extremely frequent daytime urination, urinating every 15 to 20 min without nocturnal enuresis, dysuria, and urinary tract infections [24]. Children with infrequent voiding (underactive bladder) are usually female, and they urinate just twice per day, whereas the normal frequency of urination is 4–7 times per day [25]. Overactive bladder (OAB) is a syndrome characterized by urinary urgency, associative with urinary frequency and nocturia, in the absence of any local pathological causes [19].

The presence or absence of UI in children was determined by the child’s or parents’ perception. The researcher then entered the resulting data, which included the child’s asthma status, UI status and type, age, and gender, into a checklist.

The analysis was performed by the stata software version 16. In the descriptive section of this study, frequency, percentage, mean, and standard deviation were utilized. In addition, to compare quantitative and qualitative variables, the independent t-test and analysis of variance were considered if the data had a normal distribution, whereas the Mann-Whitney U test was used if the data did not have a normal distribution. To compare two qualitative variables, Chi-Square test or Fisher’s exact test were used. Additionally, logistic regression was utilized to calculate the odds ratio.

## Results

There were 157 participants with asthma in the case group and 157 participants without asthma in the control group. The children’s average age was 8.19 ± 3.15 years (range: 3–16). There were 157 (50%) females and 157 (50%) males. 39 participants had MNE, 23 had NMNE, 77 had infrequent voiding, 21 had pollakiuria, 25 had GI, and 33 had OAB. No one had VR among the participants.

### Age and the incidence of asthma and UI

The Mann-Whitney test results presented in Table 1 indicate that the average age of patients with asthma (7.43 ± 2.89 years) was significantly lower than the patients without asthma (8.94 ± 3.22 years; p = 0.0001). GI. Moreover, In all studied urinary disorders, affected children were younger than non-affected children, with the exception of MNE, but this age difference was statistically significant only in GI patients (7 ± 3.5 years) compared to children without this disorder (8.29 ± 3.10 years; p = 0.027). It is important to note that MNE is only meaningful in children over the age of five, but because only 11 children (7 asthmatic and 4 non-asthmatic) were under the age of five and none of them had MNE, they were not eliminated from the statistical analysis of NME and they were assigned to the MNE negative group.

**Table 1:** Comparison of the mean ages of children with and without each condition

Disorder status Disorder type	Positive		Negative		P-value	OR	95% confidence interval	
	Mean age	Standard deviation	Mean age	Standard deviation			Lower	Upper
Asthma	7.43	2.89	8.94	3.22	0.0001	0.852	0.790	0.919
MNE	8.55	3.03	8.14	3.17	0.327	1.041	0.938	1.155
NMNE	6.93	2.05	8.29	3.2	0.072	0.854	0.730	1
VR	-	-	8.14	3.27	-	-	-	-
Infrequent voiding	7.60	2.81	8.38	3.23	0.084	0.921	0.845	1.003
Pollakiuria	7.42	2.63	8.24	3.18	0.293	0.915	0.785	1.065
GI	7	3.5	8.29	3.10	0.027	0.862	0.742	1.001
OAB	7.21	2.37	8.30	3.21	0.077	0.885	0.779	1.006

### The relationship between asthma and the urinary disorders

The relationship between urinary disorders and asthma is demonstrated in Table 2. The Chi-square test revealed no statistically significant relationship between asthma and MNE (p = 0.06). However, there was a significant relationship between NMNE and asthma (p = 0.017), and the odds of having NMNE in patients with asthma was 3.056 times higher than in healthy children. Furthermore, pollakiuria (p = 0.259) and GI (P = 0.532) were not significantly associated with asthma, while infrequent voiding was associated with asthma (p = 0.013) so that the incidence of infrequent urination was 1.94 times greater in asthma patients. Fisher’s exact test also showed that the likelihood of having an OAB was 5.227 times higher in patients with asthma compared to healthy children, which was a significant difference (p = 0.0001).

**Table 2:** Correlation between asthma and each urinary condition causing incontinence

Urinary disorder type	Asthma status Urinary disorder status	Positive	Negative	P-value	OR	95% confidence interval	
		Frequency (percentage%)	Frequency (percentage%)			Lower	Upper
MNE	Positive	25 (15.9)	14 (8.9)	0.06	1.934	0.965	3.879
	Negative	132 (84.1)	143 (91.1)				
NMNE	Positive	17 (10.8)	6 (3.8)	0.028	3.056	1.172	7.971
	Negative	140 (89.2)	151 (96.2)				
VR	Positive	0 (0)	0 (0)	-	-	-	-
	Negative	75 (100)	82 (100)				
Infrequent voiding	Positive	48 (30.6)	29 (18.5)	0.013	1.944	1.147	3.292
	Negative	109 (69.4)	128 (81.5)				
Pollakiuria	Positive	13 (8.3)	8 (5.1)	0.259	1.681	0.677	4.177
	Negative	144 (91.7)	149 (94.9)				
GI	Positive	14 (8.9)	11 (7)	0.532	1.299	0.571	2.958
	Negative	143 (91.1)	146 (93)				
OAB	Positive	27 (8.9)	6 (7)	0.0001	5.227	13.052	2.093
	Negative	130 (91.1)	151 (96.2)				

### The association between asthma and the urinary disorders by gender

The rate of asthma in girls was 47.8%, whereas, in boys, it was 52.2%. The likelihood of having asthma in females was 0.83 times that of boys, which was not statistically significant according to the Chi-Square test (p = 0.429).

Table 3 displays the Chi-Square test results for the connection between urinary disorders and asthma based on gender. Although, as stated, there was no significant correlation between asthma and NMNE in general, the association between these two conditions was considerable in males (p = 0.047). In contrast, NMNE was only found to be linked with asthma in females (p = 0.01). Similarly, only females showed an association between infrequent voiding and asthma (p = 0.004). OAB was the only disorder associated with asthma in both males (p = 0.001) and females (p = 0.02). GI and pollakiuria were not associated with asthma in either gender group.

**Table 3:** Correlation between asthma and each urinary condition causing incontinence by gender

Urinary disorder type	Gender	Asthma status Urinary disorder status	Positive	Negative	P-value
			Frequency (percentage%)	Frequency (percentage%)	
MNE	Female	Positive	8 (10.7)	7 (8.5)	0.650
		Negative	75 (91.5)	67 (89.3)	
	Male	Postive	17 (20.7)	7 (9.3)	0.047
		Negative	65 (79.3)	68 (90.7)	
NMNE	Female	Positive	10 (13.3)	2 (2.4)	0.01
		Negative	65 (86.7)	80 (97.6)	
	Male	Postive	7 (8.5)	4 (5.3)	0.432
		Negative	75 (91.5)	71 (94.7)	
VR	Female	Positive	0 (0)	0 (0)	-
		Negative	75 (100)	82 (100)	
	Male	Postive	-	-	-
		Negative	-	-	
Infrequent voiding	Female	Positive	28 (37.3)	14 (17.1)	0.004
		Negative	47 (62.7)	68 (82.9)	
	Male	Postive	20 (24.4)	15 (20)	0.509
		Negative	62 (75.6)	60 (80)	
Pollakiuria	Female	Positive	2 (2.7)	3 (3.7)	0.724
		Negative	73 (97.3)	79 (96.3)	
	Male	Postive	11 (13.4)	5 (6.7)	0.163
		Negative	71 (86.6)	70 (93.3)	
GI	Female	Positive	6 (8)	5 (6.1)	0.641
		Negative	69 (92)	77 (93.9)	
	Male	Postive	8 (9.8)	6 (8)	0.7
		Negative	74 (90.2)	69 (92)	
OAB	Female	Positive	15 (20)	6 (7.3)	0.02
		Negative	60 (80)	76 (92.7)	
	Male	Postive	12 (14.6)	0 (0)	0.001
		Negative	70 (85.4)	75 (100)	

## Discussion

Asthma is one of the most prevalent respiratory diseases in the world, impacting the affected children and their parents. On the other hand, non-neurogenic UI, a common complaint of children who visit health centers, has a substantial impact on children’s social and emotional development and their personality formation. Consequently, many psychological and social difficulties can be prevented by early diagnosis and prompt treatment of the aforementioned disorders. According to the findings of our study, there is a significant correlation between asthma and different urinary disorders causing incontinence.

There were 314 participants, an equal number of girls and boys. The patients’ average age was 8.19 years. When assessing children’s ages, the average age of the children with asthma and GI was less than those without these conditions. A one-unit increase in age reduced the likelihood of having asthma by 14.8% and the likelihood of GI by 13.8%. In the study conducted by Soyer et al., it was also found that asthma is correlated with the majority of dysfunctional voiding (DV) symptoms in younger children who have completed toilet training, while it is associated with some DV symptoms in older children, such as frequency and urgency [26]. Our study also revealed that children with GI were younger than children without this condition. This was also true for other urinary disorders with UI (with the exception of MNE), although the difference was not statistically significant. Therefore, the findings are consistent.

In evaluating the gender of the studied children, there was no statistically significant difference between the frequency distribution of gender in children with asthma and UI types. It would appear that asthma and UI are equally widespread among both genders. However, it has been noted in numerous studies that boys, compared to girls, are more likely to experience UI and asthma [27]. The findings of this study may contradict the findings of prior studies due to the small sample size.

The risk of MNE in children with asthma was reported to be 1.935 times higher, but this relationship between asthma and MNE was insignificant. However, a considerable association was identified between this disorder and asthma in males. Ozkaya et al. investigated the prevalence and risk variables associated with asthma and UI. They determined that the prevalence of MSE in children with asthma is much greater than in the control group. In children with asthma, pollen sensitivity, allergic rhinitis, and a high blood eosinophil count are correlated with MNE [17]. Yilmaz-Durmuş et al. demonstrated that allergic disorders (asthma, allergic rhinitis, eczema, and food allergy) are considerably greater in children with MNE than in the control group [28]. According to a study by Dahan et al., asthma is associated with early nocturnal UI, depending on the severity of sleep-related breathing disorders [29]. These studies back up the current study’s findings, and it can be proven that there is a correlation or association between respiratory disorders (including asthma) and enuresis.

NMNE had a statistically significant connection with asthma (p = 0.017). NMNE occurred 3.056 times more frequently in children with asthma. In a study of women with chronic lung disease (CLD), Button et al. determined that the presence of CLD was an independent predictor of UI in women [30]. Although this study was conducted on women and not on children, it yielded similar results, improving our findings’ precision.

Although incontinence is uncommon in pollakiuria, the symptoms of urinary irritation in this disorder have led us to look into a potential connection between pollakiuria and asthma, therefore it was investigated along with the other urinary disorders causing UI. In the evaluation of pollakiuria, asthma patients had a 1.681 times higher likelihood of developing pollakiuria, but this difference was not statistically significant (p = 0.259). However, Hou et al. found systemic or local allergic disorders, such as allergic rhinitis, allergic cough, pruritus, and an elevated total serum IgE level, are risk factors for pollakiuria in children [31]. This suggests that there may be a correlation between pollakiuria and asthma, and further research is required in this field.

In the study of GI, children with asthma were 1.29 times more likely to experience GI, though this was not statistically significant (P = 0.532). GI differs from stress incontinence, in which a small amount of urine is leaked when sneezing or coughing [32]. Stress incontinence typically affects adult females, and asthma can exacerbate its symptoms [33]. It is uncommon in children with normal neurological development [34].

There was a statistically significant increase (5.227 times) in the occurrence of OAB in asthmatic children. Zheng et al. found that OAB in children was associated with atopic symptoms including eczema, skin irritation, allergic rhinitis, allergic cough, and elevated total serum IgE level [35]. Histamine activates phospholipase C via the G protein when it binds to the H1 receptor. This raises the baseline tension in the uroepithelium, lamina propria, and detrusor layer of the bladder wall. Histamine is released when mast cells are activated, resulting in inflammation and sensitivity of the bladder. Through H1 receptors, histamine can also raise the afferent nerve’s bladder sensitivity, resulting in excessive bladder contractions [35–37]. Given that atopic asthma is the most prevalent form of asthma in children, the aforementioned mechanisms can cause OAB in children with asthma.

One of the study’s limitations is that the presence of urinary disorders was determined by outlining the disease’s symptoms and asking the children and their parents. There was no voiding chart or diary, which is required for daily practice, as well as the omission of gastrointestinal illnesses. Therefore, it is suggested that future studies investigate urinary conditions utilizing specialized questionnaires and more precise diagnostic techniques to obtain accurate results.

## Conclusions

According to the findings of our study, children with asthma are susceptible to urinary disorders such as MNE, NMNE, Infrequent voiding, and OAB. Therefore, it is necessary to examine asthmatic children for these conditions. By treating asthma, it seems that these urinary disorders can be avoided. In addition, if any of the disorders are present, their appropriate treatment can improve asthma patients’ quality of life. However, more observational studies and clinical trials are required to confirm this claim.

## Data Availability

Data will be provided by the corresponding author on request.

## List of abbreviations

MNE: monosymptomatic nocturnal enuresis
NMNE: nonmonosymptomatic nocturnal enuresis
VR: vaginal reflux
GI: giggle incontinence
OAB: overactive bladder
DV: dysfunctional voiding
